# Production of 6-l-[^18^F]Fluoro-*m*-tyrosine in an Automated Synthesis Module for ^11^C-Labeling

**DOI:** 10.3390/molecules26185550

**Published:** 2021-09-13

**Authors:** Viktoriya V. Orlovskaya, Austin S. Craig, Olga S. Fedorova, Olga F. Kuznetsova, Bernd Neumaier, Raisa N. Krasikova, Boris D. Zlatopolskiy

**Affiliations:** 1 N.P.Bechtereva Institute of the Human Brain, 197376 St. Petersburg, Russia; orlovskaya@ihb.spb.ru (V.V.O.); fedorova@ihb.spb.ru (O.S.F.); kuznets@ihb.spb.ru (O.F.K.); 2 Institute of Neuroscience and Medicine, INM-5: Nuclear Chemistry, Forschungszentrum Jülich GmbH, 52425 Jülich, Germany; a.craig@hzdr.de (A.S.C.); boris.zlatopolskiy@uk-koeln.de (B.D.Z.); 3 Institute of Radiochemistry and Experimental Molecular Imaging, University Clinic Cologne, 50937 Cologne, Germany; 4 Max Planck Institute for Metabolism Research, 50931 Cologne, Germany

**Keywords:** fluorine-18, radiofluorination, Cu-mediated, alcohol-enhanced, 6-l-[^18^F]fluoro-*m*-tyrosine, automated synthesis

## Abstract

6-l-[^18^F]Fluoro-*m*-tyrosine (6-l-[^18^F]FMT) represents a valuable alternative to 6-l-[^18^F]FDOPA which is conventionally used for the diagnosis and staging of Parkinson’s disease. However, clinical applications of 6-l-[^18^F]FMT have been limited by the paucity of practical production methods for its automated production. Herein we describe the practical preparation of 6-l-[^18^F]FMT using alcohol-enhanced Cu-mediated radiofluorination of Bpin-substituted chiral Ni(II) complex in the presence of non-basic Bu_4_ONTf using a volatile *i*PrOH/MeCN mixture as reaction solvent. A simple and fast radiolabeling procedure afforded the tracer in 20.0 ± 3.0% activity yield within 70 min. The developed method was directly implemented onto a modified TracerLab FX C Pro platform originally designed for ^11^C-labeling. This method enables an uncomplicated switch between ^11^C- and ^18^F-labeling. The simplicity of the developed procedure enables its easy adaptation to other commercially available remote-controlled synthesis units and paves the way for a widespread application of 6-l-[^18^F]FMT in the clinic.

## 1. Introduction

Assessing the metabolic activity of l-aromatic amino acid decarboxylase (AADC) [1] with 6-l-[^18^F]fluoro-3,4-dihydroxyphenylalanine (6-l-[^18^F]FDOPA) PET is widely used for the diagnosis and staging of Parkinson’s disease (PD). In patients with PD, the activity of AADC in the striatum is reduced to 5–20% of normal levels before cardinal motor symptoms become apparent [2]. However, kinetic modeling and quantification of 6-l-[^18^F]FDOPA uptake is complicated by its peripheral metabolism mediated by catechol *O*-methyltransferase (COMT). The resulting metabolite, 3-*O*-methyl-[^18^F]FDOPA, crosses the blood–brain barrier (BBB) and contributes to an undesirable background uptake into the brain, thereby reducing the signal-to-background ratio for 6-l-[^18^F]FDOPA. To address this problem, an alternative non-catechol radiotracer 6-l-[^18^F]fluoro-*m*-tyrosine (6-l-[^18^F]FMT) has been developed as a suitable substrate for AADC, which is simultaneously no substrate for COMT-mediated *O*-methylation [3,4]. Due to increased signal-to-noise ratio in the regions of interest, 6-l-[^18^F]FMT demonstrates higher sensitivity compared to 6-l-[^18^F]FDOPA and enables a better quantification of AADC levels and activities [5,6,7,8]. While 6-l-[^18^F]FMT PET has been widely used in rodents and nonhuman primate models of PD [4,6,9,10], only a few applications in human studies have been reported [5,8,11,12]. The clinical application of this radiotracer is significantly limited by the absence of practical automated production methods that could provide the tracer in Curie-level amounts.

Until recently, 6-l-[^18^F]FMT has been most commonly prepared via electrophilic fluorodemetalation of a commercially available stannylated precursor (*N*-trifluoroacetyl-3-acetoxy-6-trimethylstannyl-l-phenylalanine ethyl ester) with [^18^F]F_2_. The subsequent purification of the radiolabeled intermediate by flash chromatography on alumina, followed by its hydrolysis and semi-preparative HPLC purification, afforded 6-l-[^18^F]FMT in 17% radiochemical yield [13] (RCY) [14]. Comparable RCYs were reported if *bis*-*N*,*O*-Boc protected precursor was applied [15]. Despite the apparent simplicity and adaptability to automated synthesis modules, electrophilic radiofluorination methods have several limitations, including difficulties to manipulate gaseous [^18^F]F_2_, insufficient productivity of gas targets and low molar activities (A_m_) of the final product owing to unavoidable dilution with ^19^F [16].

In contrast, radiosyntheses with easily accessible no-carrier-added nucleophilic [^18^F]fluoride lack these disadvantages and could furnish large quantities of radiofluorinated tracers. Unfortunately, preparation of ^18^F-labeled aromatic amino acids ([^18^F]FAAs) lacking an electron-withdrawing group in the phenyl ring using conventional S_n_Ar radiofluorination is challenging. Therefore, ^18^F-fluorination strategies typically require multistep syntheses starting from ^18^F-labeling of substituted benzaldehyde with an appropriate leaving group, followed by a series of transformations to build up the target radiolabeled aromatic amino acid. The most successful phase-transfer catalytic (PTC) asymmetric synthesis [17,18,19], particularly valuable for 6-l-[^18^F]FDOPA, involved five synthesis steps. The key step consisting of alkylation of a suitable glycine equivalent in the presence of a chiral phase transfer catalyst could be carried out with high stereoselectivity resulting in high enantiomeric purity of the desired [^18^F]FAA tracer. This approach has also been recently applied for the production of 6-l-[^18^F]FMT [10]. Despite the high enantiomeric purity of the resulting products and reasonable RCYs of up to 35%, there are still concerns about the complexity of this procedure and in some cases the limited stability of starting materials [20].

Alternatively, a three-step nucleophilic labeling method utilizing isotopic ^19^F/^18^F exchange in a formyl-activated aromatic amino acid derivative, followed by Baeyer–Villiger oxidation and acid hydrolysis, has been disclosed recently [21]. This method, originally developed for the synthesis of 6-l-[^18^F]FDOPA, has been evaluated on a small scale for the preparation of 6-l-[^18^F]FMT [22]. Fair RCYs of 8–13% and cumbersome precursor synthesis limited the practical applications of this method.

**Figure 1:**
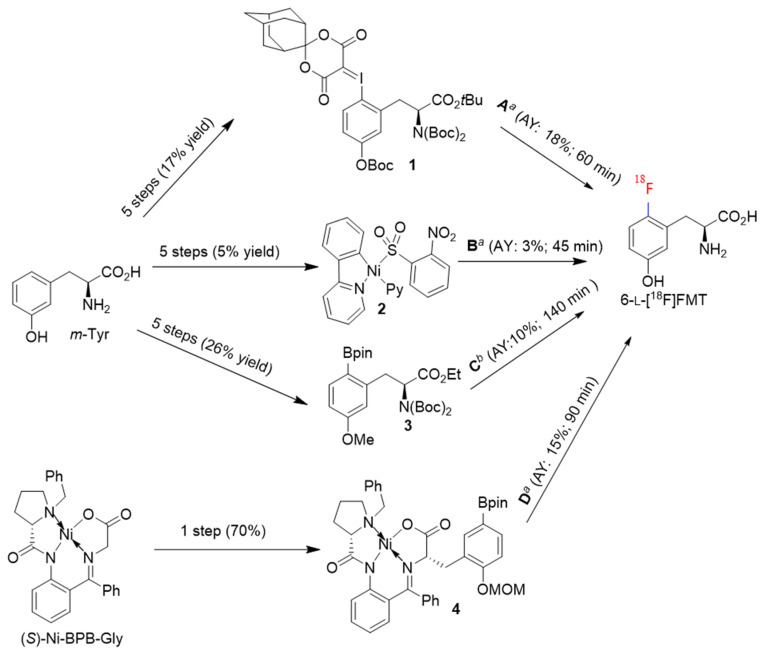
Preparation of 6-l-[^18^F]FMT via late-stage radiofluorination with [^18^F]fluoride. Radiolabeling conditions: (**A**) (i) azeotropic drying of [^18^F]Et_4_NF/Et_4_NHCO_3_ (×3), (ii) DMF, 120 °C, 20 min [24]; (**B**) [^18^F]KF/K_2_CO_3_/18-crown-6, 1,1’-(phenyl-λ^3^-iodanediyl)bis(4-methoxypyridin-1-ium) trifluoromethanesulfonate, MeCN, r.t., 5 min [25]; (**C**) (i) azeotropic drying of [^18^F]KF/K_2_C_2_O_4_/K2.2.2 (×5), (ii) Cu(py)_4_(OTf)_2_, DMF, 120 °C, 20 min, air [29]; (**D**) [^18^F]Et_4_NF/Et_4_NHCO_3_, Cu(py)_4_(OTf)_2_, *n*BuOH/DMA, 110 °C, 15 min, air [34]. Procedures (**B**) and (**D**) avoid any azeotropic drying steps. *^a^*—manual synthesis; *^b^*—synthesis in a remotely controlled unit. AY—activity yield of the HPLC purified 6-l-[^18^F]FMT [13]; r.t.—room temperature.

The most recent approaches for ^18^F-labeling of non-activated aromatics rely upon “late-stage radiofluorinations” using a two-step sequence of reactions: radiofluorination of appropriate precursors (iodonium salts, iodonium ylides, nickel complexes, organoborons or stannanes) followed by acid hydrolysis [23]. Among the methods reported, radiofluorination of the appropriate spirocyclic iodonium(III)ylide **1 **(Figure 1A) afforded the radiotracer in 18% activity yield (AY) [13] within a synthesis time of ca. 60 min [24]. However, this approach was applied only on a small scale (20–60 MBq) and was not further translated into large-scale automated production. Alternatively, radiofluorination of the appropriate Ni complex precursor **2 **(Figure 1B) in the presence of a hypervalent iodine(III) salt afforded 6-l-[^18^F]FMT in 5% RCY within 60 min [25]. Unfortunately, a pronounced instability of the applied oxidant precludes wider application of this protocol. 

Cu-mediated radiofluorination of pinacol arylboronates (ArylBPin) [26] using commercially available Cu(py)_4_(OTf)_2_ has been found to be particularly advantageous for the preparation of a wide range of radiotracers [27]. The selection of the salt (or PTC/base combination) for ^18^F^–^ preprocessing is known to have a profound impact on the outcome of base-sensitive Cu-mediated radiofluorinations [28]. Thus, in the first adaptation of this approach for the automated preparation of 6-l-[^18^F]FMT from precursor **3** (Figure 1C) [29], the conventional [^18^F]KF/K_2_CO_3_/Kryptofix 222 (K_222_) complex was replaced with a less basic [^18^F]KF/K_2_C_2_O_4_/K_2_CO_3_/K_222_ combination. Disadvantageously, boiling concentrated HI was applied for the deprotection step. This reagent is extremely corrosive and requires replacement of capillary connections and valves after each production. The remotely controlled synthesis on a Synthra platform afforded 6-l-[^18^F]FMT in 10% AY within 140 min (Table 1, entry 1). Additionally, repetitive azeotropic drying with MeCN was required to completely remove water. This time-consuming procedure is associated with radioactivity losses owing to radioactive decay and unavoidable heating-induced adsorption of [^18^F]fluoride on reactor walls. In recent years, several groups suggested protocols obviating azeotropic drying for Cu-mediated fluorination which apply solutions of weakly or non-basic organic salts in suitable protic or aprotic solvents [30,31,32]. Among them, the procedure for alcohol-enhanced Cu-mediated radiofluorination, which consists of the direct elution of ^18^F^−^ with tetraalkylammonium or phosphonium salts in *n*BuOH into a solution of Cu mediator and precursor in DMA followed by brief heating of the resulting mixture [30] under air, has found broad application for radiolabeling of various substrates. The modification of this procedure (elution of ^18^F^−^ with methanolic Et_4_NHCO_3_ followed by removal of low boiling MeOH and Cu-mediated radiofluorination in *n*BuOH/DMA medium under air) [33] was applied for the preparation of 6-l-[^18^F]FMT from the Ni complex **4** (Figure 1D) [34]. This crystalline precursor, which contains a Ni-BPB auxiliary as an easily removable dual-protecting group functionality, is readily accessible on a multi-gram scale and can be handled without special precautions. Less corrosive 37% HCl was used for the decomposition of the radiolabeled Ni complex intermediate. Whereas the procedure was highly efficient for the manual synthesis of the radiolabeled amino acid (15 ± 3% AY), its implementation for the automated production of 6-l-[^18^F]FMT was less satisfactory (6 ± 5% RCY) (Table 1, entry 2).

In this work, we describe a novel procedure for the automated production of 6-l-[^18^F]FMT based on the modified protocol for alcohol-enhanced Cu-mediated radiofluorination. The latter employs Bu_4_NOTf as non-basic PTC and low-boiling *i*PrOH/MeCN mixture as reaction medium. 

## 2. Results and Discussion

### 2.1. Optimization of the Manual 6-l-[^18^F]FMT Radiosynthesis

**Scheme 1:**
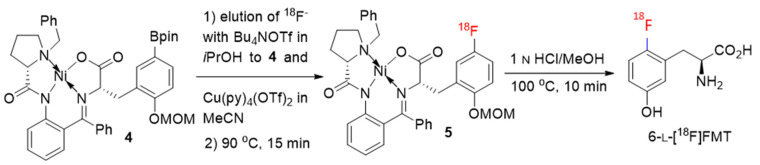
Radiosynthesis scheme for 6-l-[^18^F]FMT by Cu-mediated radiofluorination of **4**.

6-l-[^18^F]FMT was produced as outlined in Scheme 1.

Based on our previous findings [32], we tested to replace the weak base Et_4_NHCO_3_ in high boiling *n*BuOH with non-basic Bu_4_NOTf in lower boiling *i*PrOH for elution of [^18^F]fluoride. Application of the “back-flushing” protocol [25] enabled >90% ^18^F^–^ recovery (*n* > 30). [^18^F]Fluoride was directly eluted into a solution of **4** and Cu mediator, avoiding any evaporation steps.

Initially, the radiofluorination step was carried out in *i*PrOH/DMA using 15 µmol precursor and 7.5 µmol Cu mediator as described previously [32]. Despite high ^18^F incorporation rates (>80% by radioTLC), we were faced with incomplete hydrolysis of the intermediary radiolabeled complex **5** if HCl was directly added to the reaction mixture. Furthermore, concentrated HCl was required to achieve reasonable (>50%) RCYs of 6-l-[^18^F]FMT (determined by HPLC). 37% HCl is rather corrosive and not well compatible with the components of automated modules. Concentration of the reaction mixture could simplify the hydrolysis/deprotection step and enable the application of diluted HCl. In order to facilitate solvent removal, we replaced DMA with highly volatile MeCN. This modification required a reassessment of radiolabeling conditions. Accordingly, the highest RCYs of **5** were obtained using 10 µmol of radiolabeling precursor and 16 µmol Cu(py)_4_(OTf)_2_, respectively, in 27% MeCN in *i*PrOH (1.1 mL). After the reaction mixture was heated to 90 °C for 15 min, all volatiles were removed at 100 °C under a flow of N_2_ within 3–4 min. Finally, the product was released using a mixture of aqueous HCl/MeOH; MeOH was added to homogenize the reaction mixture.

**Figure 2:**
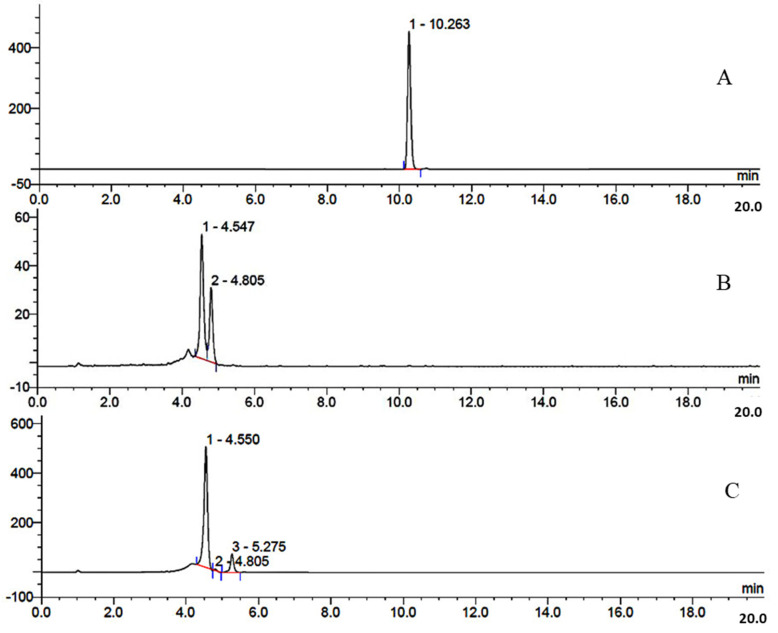
Radio-HPLC analysis of the reaction mixture: (**A**) radiofluorination of **4** (preparation of **5**); (**B**) hydrolysis of **5** with 1.5 n HCl in 50% MeOH; (**C**) hydrolysis of **5** with 0.5 n HCl in 50% MeOH. HPLC conditions: column: XBridge^®^ C18 5 µm, 150 × 4.6 mm (Waters, Millford, CT, USA); gradient: 0–2 min: 2% MeCN (0.1% TFA), 2–13 min: 2–90% MeCN (0.1% TFA); flow rate: 2.0 mL/min; R_t_ of **5**: 10.3 min; R_t_ of 6-l-[^18^F]FMT: 4.6 min.

Several conditions for decomposition of **5** were tested. Heating of 1.5 n HCl in 50% MeOH (1 mL) led to the fast and complete hydrolysis of the ^18^F-fluorinated intermediate. Unfortunately, up to 36% of an unidentified labeled byproduct was observed in this case (Figure 2B, Block **I** in Supplementary Materials). Application of 0.5 n HCl in the same solvent allowed us to suppress the formation of this byproduct to 12% (Figure 2C). Lower acid concentration was not sufficient to completely deprotect/decompose **5**. Notably, 6-l-[^18^F]FMT was efficiently separated from the impurity by semi-preparative HPLC.

### 2.2. Automated Production of 6-l-[^18^F]FMT

One of the decisive factors for a fast, successful implementation of radiosynthesis into a remotely controlled module is the simplicity of the initial preparation procedure. Accordingly, we implemented a very simple setup for the preparation of 6-l-[^18^F]FMT by Cu-mediated radiofluorination: pre-loading of the reactor vial with a solution of radiolabeling precursor and Cu mediator in the reaction solvent followed by the elution of [^18^F]fluoride directly into the reaction vessel, heating of the reaction mixture, and hydrolysis of the radiolabeled intermediate **5** in the same vessel. For the purpose of this study we modified a TRACERlab FX C Pro synthesis module (GE Healthcare, Waukesha, WI, USA) developed for ^11^C-labeling using gaseous radiolabeled precursors, such as [^11^C]CO_2_ and [^11^C]CH_4_, which is underutilized in our lab owing to the steadily increasing demand of ^18^F-labeled compounds compared to ^11^C-labeled tracers. Accordingly, the modified module configuration with additional components (valves, luer adapters, etc.) enabled us to carry out [^18^F]fluoride preprocessing. The module was originally equipped with a small 2 mL reaction vessel, compared with the standard 20 mL reactor in modules commonly used for nucleophilic radiofluorinations such as the TRACERlab FX N Pro. Therefore, the amounts of solvents had to be adjusted to carry out both fluorination and hydrolysis in the small reactor. All parameters optimized for the manual radiosynthesis were used in the automated tracer production without further modifications. Notably, radiofluorination proceeds under nitrogen instead of air usually applied for Cu-mediated ^18^F-fluorination of boronate and stannyl substrates [26]. This could be advantageous if the module is used also for the production of other PET tracers which require inert conditions.

For the isolation of 6-l-[^18^F]FMT, the HPLC system, originally available on the GE Tracerlab FX C Pro module, was used without any modifications.

**Figure 3:**
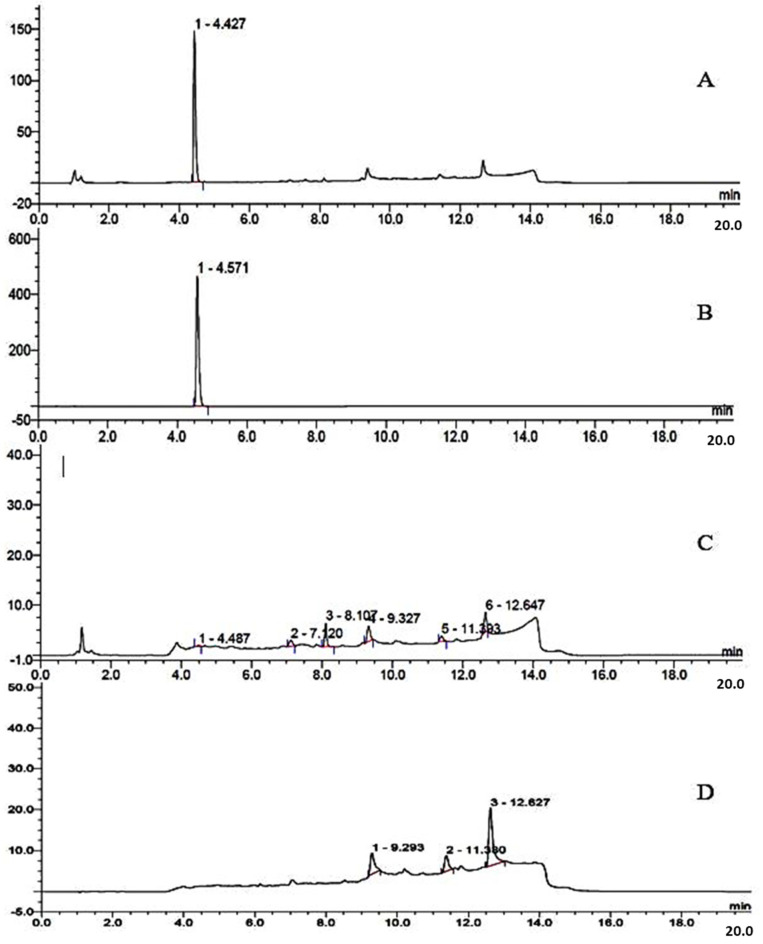
Quality control of 6-l-[^18^F]FMT: (**A**) 6- dl-FMT, UV trace (λ = 254 nm); (**B**) 6-l-[^18^F]FMT, radioactivity trace; (**C**) 6-l-[^18^F]FMT, UV trace (λ = 254 nm); (**D**) injection of the starting eluent (2% MeCN (0.1% TFA)), UV trace (λ = 254 nm); HPLC conditions: column: XBridge^®^ C18 5 µm, 150 × 4.6 mm (Waters, Millford, CT, USA); gradient: 0–2 min: 2% MeCN (0.1% TFA), 2–13 min: 2–90% MeCN (0.1% TFA); flow rate: 2.0 mL/min; R_t_ of 6-l-[^18^F]FMT: 4.6 min; (**D**) blank UV chromatogram at 254 nm. Please, notice that the diffuse peak at 3.8–14.0 min and peaks at 3.9; 4.5; 7.2; 8.1; 9.3; 11.4; and 12.6 min (UV 254 nm) are also visible if the starting eluent is injected under the same chromatographic conditions.

**Figure 4:**
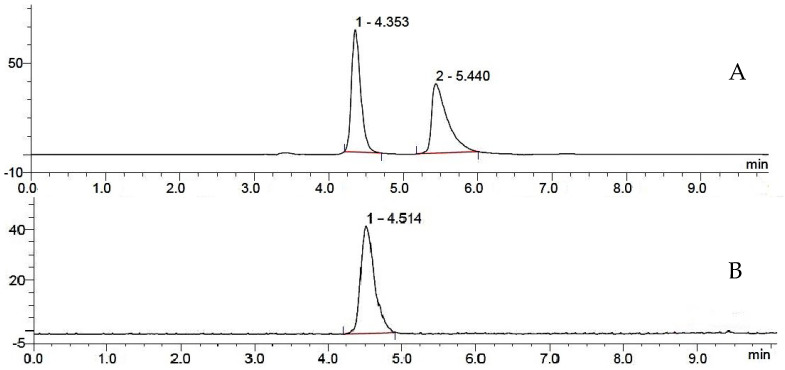
Determination of enantiomeric purity of 6-l-[^18^F]FMT by chiral HPLC: (**A**) 6-dl-FMT, UV trace (λ = 254 nm); peak 1-6-l-FMT; peak 2-6-d-FMT; (**B**) 6-l-[^18^F]FMT, radioactivity trace. HPLC conditions: column Chirobiotic T 150 × 4.6 mm (Astec, Whippany, NJ, USA); eluent: 10% EtOH in 0.1% Et_3_NOAc; flow rate: 1.0 mL/min.

The AY of 6-l-[^18^F]FMT was 20 ± 3% (*n* = 3), within a total synthesis time of 70 min comprising purification and formulation. The radiotracer was obtained in a radiochemical purity (Figure 3B, Block **II** in Supplementary Materials) and enantiomeric purity (Figure 4B, Block **III** in Supplementary Materials) of >99% and molar activities of 125–250 GBq/µmol (EOS) (Block **IV** in Supplementary Materials). The residual copper and nickel contents, measured by ICP/MS, were less than 0.05 ppm and 0.004 ppm, respectively. These values were below any level of concern according to the ICH Guideline of Elemental Impurities (Q3D) [35]. The amount of residual Bu_4_N salts was below the detection limit of the applied HPLC method. In the corresponding UV chromatogram, no peak with a R_t_ of 5.6 min, which corresponds to Bu_4_N^+^, was observed (Figure 3C).

## 3. Experimental

### 3.1. Materials and Methods

Unless otherwise stated, reagents and solvents were commercially available and used without further purification. Anhydrous acid-free MeCN (max 10 ppm H_2_O) was purchased from Kriochrom, Russia. The precursor for 6-l-[^18^F]FMT, (*S*,*S*)-Ni-BPB-2-Bpin-5-MOMO-Phe (**4**, Figure 1), was prepared according to [34]; the reference standard of 6-dl-FMT was obtained from Merck. Deionized water (18.2 MΩ*cm) from an in-house Millipore Simplicity purification system was used for the preparation of all aqueous solutions. [^18^O]H_2_O (97% enrichment) was purchased from Global Scientific Technologies, Russia. Sep-Pak Accell Plus QMA Plus Light Cartridges (130 mg) were acquired from Waters Corporation, Millford, CT, USA and were conditioned with 10 mL of 0.05 m NaHCO_3_ followed by 10 mL H_2_O before application.

Radio-TLC analyses were carried out on silica gel plates (60 F254, Merck or Sorbfil, Lenchrom, Russia); radioactivity distribution was determined using radioTLC scanner miniGita (Raytest, Straubenhardt, Germany) or Scan-RAM radioTLC scanner controlled by the chromatography software package Laura for PET (LabLogic, Sheffield, UK). An aliquot of the crude reaction mixture (2–3 μL) was applied onto a TLC plate, and the plate was then developed in EtOAc/CHCl_3_/AcOH (4/1/1); the R_f_ values for [^18^F]F^–^ and ^18^F-labeled intermediate **5** amounted to 0.05 and 0.83, respectively. The radiochemical conversion (RCC) was defined as the ratio of the product peak area to total peak area on the TLC; the RCC value was not corrected for decay. Radiochemical purity of 6-l-[^18^F]FMT (R_f_ 0.70) was determined using MeOH/AcOH (9/1) as an eluent.

The analytical HPLC system ICS-5000 (Dionex, Sunnyvale, CA, USA) consisted of a gradient pump, Rheodyne-type injector with a 20 μL loop and variable wavelength UV absorbance detector (set to 254 nm) connected in series with a radiodetector (Carrol and Ramsey Associates model 105-S). HPLC analytics: column: XBridge^®^ C18 5 µm, 150 × 4.6 mm (Waters, Millford, CT, USA); gradient: 0–2 min: 2% MeCN (0.1% TFA), 2–13 min: 2–90% MeCN (0.1% TFA); flow rate: 2.0 mL/min.

The UV and radioactivity detectors were connected in series resulting in a delay of 0.1 min. The R_t_ values of **5** and 6-l-[^18^F]FMT were 10.3 and 4.5 min, respectively. Bu_4_NOTf content in the final product was determined using the same conditions; R_t_ (Bu_4_N^+^): 5.6 min. Enantiomeric purity of 6-l-[^18^F]FMT was determined using the following conditions: column: Chirobiotic T 150 × 4.6 mm (Astec, Whippany, NJ, USA); eluent: 10% EtOH in 0.1% Et_3_NOAc; flow rate: 1.0 mL/min. R_t_ values for l- and d-isomers of FMT were 4.3 and 5.4 min, respectively. Ni and Cu content in the final formulation was determined by ICP-MS using ICP Optical Emission Spectrometer Varian 725-ES (Varian, Palo Alto, CA, USA). 

6-l-[^18^F]FMT was isolated using the HPLC package available on GE Tracerlab FX C Pro module (GE Healthcare, Waukesha, WI, USA), consisting of a SYCAM S1122 pump, UV detector KNF, LAB LABOPORT, 2 mL injection loop and β-radioactivity flow detector. Conditions: column: C_18_ amide, 250 × 10 mm, 5 µm (Supelco, Bellefonte, PA, USA) equipped with a guard column (Security Guard Cartridge AJO-8327-S in Guard Cartridge Holder KJO-4282, Phenomenex, Torrance, CA, USA); eluent: 0.1% AcOH; flow rate: 4 mL/min; R_t_ for 6-l-[^18^F]FMT: ~12 min.

### 3.2. Production of [^18^F]Fluoride

[^18^F]Fluoride was produced via the ^18^O(p,n)^18^F nuclear reaction by irradiation of [^18^O]H_2_O (97% enrichment, Global Scientific Technologies, Sosnovyj Bor, Russia) in a Ag target (1.4 mL) with 16.4 MeV protons at a PETtrace 4 cyclotron (GE Healthcare, Uppsala, Sweden). The irradiated [^18^O]H_2_O was transferred from the target using a flow of helium and loaded onto a Sep-Pak Accell Plus QMA Plus Light Cartridge (130 mg). The luer-lock fitting of the cyclotron delivery line was connected to the male side of the cartridge.

### 3.3. Manual Radiosynthesis of 6-l-[^18^F]FMT

A QMA cartridge loaded with [^18^F]fluoride (7–15 GBq) was washed with *i*PrOH (3 mL) from the male side and dried with nitrogen gas for 2 min. Afterwards, ^18^F^−^ was eluted from the resin backwards relative to the loading direction with a solution of Bu_4_NOTf (5 mg, 12.5 µmol) in *i*PrOH (0.8 mL) into the 2 mL reaction vessel prefilled with a solution of **4** (7.7 mg, 10 µmol) and Cu(py)_4_(OTf)_2_ in MeCN (0.3 mL). The reaction mixture was stirred at 90 °C for 15 min. Thereafter, volatiles were evaporated at 80 °C under N_2_ flow and the reaction vessel was cooled down to 40 °C. A total of 0.5 n HCl in 50% MeOH (1 mL) was added and the mixture was stirred at 100 °C for 10 min. When hydrolysis was completed (HPLC control), volatiles were removed at 120 °C under N_2_ flow. The reaction vessel was cooled down to 50 °C, MeOH (0.5 mL) was added and the resulting solution was transferred into the reaction vessel containing 0.1% AcOH (1.4 mL) of a GE Tracerlab FX C Pro module. The content of the vessel (total volume about 1.8–1.9 mL) was loaded into the HPLC loop. The 6-l-[^18^F]FMT (R_t_ 12 min) fraction (4–5 mL) was collected through a 0.22 μm filter (Millipore, Burlington, MA, USA) in a vented sterile vial prefilled with sterile saline (5 mL).

### 3.4. Automated Radiosynthesis of 6-l-[^18^F]FMT

**Figure 5:**
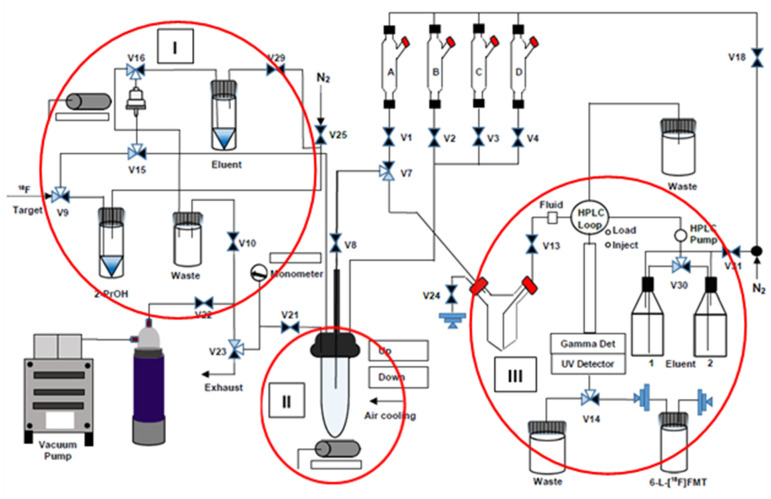
Schematic diagram of the GE TRACERlab FX-C Pro module modified for the preparation of 6-l-[^18^F]FMT. **A**: empty vessel; **B**: 0.5 n HCl in 50% MeOH (1 mL); **C**: MeOH (0.5 mL); **D**: 0.1% AcOH (1.5 mL); **RV**: **4** (7.7 mg, 10 µmol) and Cu(py)_4_(OTf)_2_ (11 mg, 16 µmol) in MeCN (0.3 mL); eluent vial: Bu_4_NOTf (5 mg, 12.5 µmol) in *i*PrOH (0.8 mL). RV—reaction vessel (2 mL).

The commercially available synthesis module TRACERlab FX C Pro (GE Healthcare, Waukesha, WI, USA) was employed after some modifications. This module, primarily designed for ^11^C-labeling, was modified for radiofluorination with regard to both hardware and software (sequence timings). The modified module, described in Figure 5, was composed of three functional blocks: Block **I**: preprocessing of [^18^F]fluoride; Block **II**: radiosynthesis (radiofluorination and hydrolysis); Block **III**: HPLC purification and formulation. 

The most significant modification of the module configuration was introduced in Block **I** (Figure 5) for [^18^F]fluoride preprocessing. For this purpose, additional valves V9, V15 and V16 were installed and integrated into the control program along with two Wheaton vials (5 mL) with screw caps prefilled with *i*PrOH and QMA eluent (Bu_4_NOTf in *i*PrOH). Nitrogen gas for the reagent transfer was supplied via V25 and V29 valves.

Only minor configurational changes were made on the side of the reaction vessel (RV) and HPLC purification system (Blocks **II** and **III**). The original 2 mL RV was used for the radiofluorination and hydrolysis/deprotection steps. The amounts of solvents and solutions used in every synthesis step were adjusted to this small volume RV. Synthesis steps and their operation modes are summarized in Table 2.

## 4. Conclusions

The preparation of 6-l-[^18^F]FMT using Cu-mediated radiofluorination of Bpin-substituted chiral Ni complex **4** was successfully implemented onto a TRACERlab FX C Pro radiosynthesis module usually designed for ^11^C-labeling. This easy switch between ^18^F and ^11^C labeling could be of high interest owing to the steadily increasing demand for radiofluorinated, compared to ^11^C-labeled, tracers. Application of non-basic Bu_4_NOTf for ^18^F-elution and low boiling mixture *i*PrOH and MeCN as reaction medium enabled the fast and efficient production of 6-l-[^18^F]FMT without the need for any intermediary purification and with the single evaporation step. The simplicity of the developed procedure allows its easy adaptation to other commercially available remote-controlled synthesis units and paves the way for widespread application of this important PET radiotracer.

## Figures and Tables

**Table 1:** Automated production of 6-l-[^18^F]FMT by Cu-mediated radiofluorination of ArylBPin precursors.

Nr.	Precursor	Fluorination Conditions	Hydrolysis/Deprotection Conditions	Precursor/Cu-Catalyst (µmol)	AY (%)	Synthesis Time (min);Automation Mode	Reference
1	3	K_222_/K_2_C_2_O_4_/ K_2_CO_3_ Cu(py)_4_(OTf)_2_, DMF,120 °C, 20 min, air	57% HI, 150 °C, 10 min	20/22	10	140; Synthra	[29]
2	4	Et_4_NHCO_3_, Cu(py)_4_(OTf)_2_, *n*BuOH/DMA 110 °C, 15 min, air	12 n HCl 110 °C, 10 min	10/20	6 ± 5	90; Semi-automated	[34]
3	4	Bu_4_NOTf, Cu(py)_4_(OTf)_2_, *i*PrOH/MeCN 90 °C, 15 min, N_2_	1 n HCl/MeOH 100 °C, 10 min	10/16	20 ± 3	70; TracerLab FX C Pro (modified)	This work

**Table 2:** Process sequence for the automated synthesis of 6-l-[^18^F]FMT using the modified GE Tracerlab FX C Pro module (Figure 5).

Entry	Process	Activated Path/Function
Block **I**		
1	Loading of [^18^F]fluoride onto the QMA cartridge	V9-V15-QMA-V16-waste bottle-V10-V22-Vac
2	Washing of QMA cartridge with *i*PrOH (3 mL)	V25-V29-V9-V15-QMA-V16-waste bottle-V10-V22-Vac
3	Elution of [^18^F]fluoride from the cartridge into the RV.	V25-V29-eluent vial-V16-QMA-V15-RV-V21-V23
Block **II**		
4	Radiofluorination, RV, 90 °C, 15 min stirring	-
5	Removal of volatiles at 80 °C under N_2_ flow	V18-V1-V7-V8-RV-V21-V23
6	Transfer of 0.5 n HCl in 50% MeOH from B to RV	V18-V2 -RV-V21-V23
7	Hydrolysis, RV, 100 °C, 10 min	-
8	Removal of volatiles at 120 °C under N_2_ flow	V18-V1-V7-V8-RV-V21-V23
9	Transfer of MeOH from C to RV, stirring	V18-V3-RV-V21-V23
10	Transfer of 0.1% AcOH from D to RV, stirring	V18-V4-RV-V21-V23
Block **III**		
11	Solution transfer from RV into the V-shaped vessel	V2-RV-V8-V7-V24-V-shaped vessel
12	Loading the content of V-shaped vessel into HPLC loop	V2-RV-V8-V7-V24-V-shaped vessel-V13, Load/Inject
13	Collection of the radioactive product in the product vial	V14, manual operation

## Data Availability

Not applicable.

## Supplementary Materials

The following are available online: radio-HPLC chromatograms, Block **I**: Radiofluorination of Bpin-substituted chiral Ni complex **4** and hydrolysis of radiolabeled intermediate [^18^F]**5**: radio-HPLC traces of the crude reaction mixtures, Block **II**: Quality control of 6-l-[^18^F]FMT (HPLC traces), Block **III**: Control of enantiomeric purity of 6-l-[^18^F]FMT, Block **IV**: Determination of molar activity (A_m_) of 6-l-[^18^F]FMT.

## Sample Availability

Samples are not available due to being perishable goods.

## References

[1] Kilbourn M.R. (2021). ^11^C- and ^18^F-Radiotracers for In Vivo Imaging of the Dopamine System: Past, Present and Future. Biomedicines.

[2] Nagatsu T., Sawada M. (2007). Biochemistry of post-mortem brains in Parkinson’s disease: Historical overview and future prospects. J. Neural Transm. Suppl..

[3] DeJesus O.T., Sunderland J.J., Nickles J.R., Mukherjee J., Appelman E.H. (1990). Synthesis of radiofluorinated analogs of *m*-tyrosine as potential l-dopa tracers via direct reaction with acetylhypofluorite. Int. J. Rad. Appl. Instrum. A.

[4] Barrio J.R., Huang S.-C., Yu D.-C., Melega W.P., Quintana J., Cherry S.R., Jacobson A., Namavari M., Satyamurthy N., Phelps M.E. (1996). Radiofluorinated L-*m*-tyrosines: New in-vivo probes for central dopamine biochemistry. J. Cereb. Blood Flow Metab..

[5] Nahmias C., Wahl L., Chirakal R., Firnau G., Garnett E.S. (1995). A probe for intracerebral aromatic amino-acid decarboxylase activity: Distribution and kinetics of [^18^F]6-fluoro-L-*m*-tyrosine in the human brain. Mov. Disord..

[6] Doudet D.J., Chan G.L.-Y., Jivan S., DeJesus O.T., McGeer E.G., English C., Ruth T.J., Holden J.E. (1999). Evaluation of Dopaminergic Presynaptic Integrity: 6-[^18^F]Fluoro-L-Dopa Versus 6-[^18^F]Fluoro-L-*m*-Tyrosine. J. Cereb. Blood Flow Metab..

[7] Eberling J.L., Bankiewicz K.S., O’Neil J.P., Jagust W.J. (2007). PET 6-[^18^F]fluoro-L*-m*-tyrosine Studies of dopaminergic function in human and nonhuman primates. Front. Hum. Neurosci..

[8] Li C.T., Palotti M., Holden J.E., Oh J., Okonkwo O., Christian B.T., Bendlin B.B., Buyan-Dent L., Harding S.J., Stone C.K. (2014). A dual-tracer study of extrastriatal 6-[^18^F]fluoro-*m*-tyrosine and 6-[^18^F]fluoro-L-dopa uptake in Parkinson’s disease. Synapse.

[9] DeJesus O., Endres C.J., Shelton S.E., Nickles R.J., Holden J.E. (1997). Evaluation of fluorinated *m*-tyrosine analogs as PET imaging agents of dopamine nerve terminals: Comparison with 6-fluorodopa. J. Nucl. Med..

[10] Becker G., Bahri M.A., Michel A., Hustadt F., Garraux G., Luxen A., Lemaire C., Plenevaux A. (2017). Comparative assessment of 6-[^18^F]fluoro-L-*m*-tyrosine and 6-[^18^F]fluoro-L-dopa to evaluate dopaminergic presynaptic integrity in a Parkinson’s disease rat model. J. Neurochem..

[11] Gallagher C.L., Christian B.T., Holden J.E., Dejesus O.T., Nickles R.J., Buyan-Dent L., Bendlin B.B., Harding S.J., Stone C.K., Mueller B. (2011). A within-subject comparison of 6-[^18^F]fluoro-*m*-tyrosine and 6-[^18^F]fluoro-L-dopa in Parkinson’s disease. Mov. Disord..

[12] Asari S., Fujimoto K., Miyauchi A., Sato T., Nakano I., Muramatsu S. (2011). Subregional 6-[^18^F]fluoro-*m*-tyrosine uptake in the striatum in Parkinson’s disease. BMC Neurol..

[13] Coenen H.H., Gee A.G., Adam M., Antoni G., Cutler C.S., Fujibayashi Y., Jeong J.M., Mach R.H., Mindt T.L., Pike V.W. (2018). Open letter to journal editors on: International consensus radiochemistry nomenclature guidelines. J. Label. Comp. Radiopharm..

[14] Namavari M., Satyamurthy N., Phelps M.E., Barrio J.R. (1993). Synthesis of 6-[^18^F] and 4-[^18^F]fluoro-L-*m*-tyrosines via regioselective radio-fluorodestannylation. Appl. Radiat. Isot..

[15] Van Brocklin H.F., Blagoev M., Hoepping A., O’Neil J.P., Klose M., Schubiger P.A., Ametamey S. (2004). A new precursor for the preparation of 6-[^18^F]Fluoro-l-*m*-tyrosine ([^18^F]FMT): Efficient synthesis and comparison of radiolabeling. Appl. Radiat. Isot..

[16] Wagner M., Wuest F. (2019). The Radiopharmaceutical chemistry of fluorine-18: Electrophilic fluorinations. Radiopharm. Chem..

[17] Libert L.C., Franci X., Plenevaux A.R., Ooi T., Maruoka K., Luxen A.J., Lemaire C.F. (2013). Production at the Curie level of no carrier-added 6-^18^F-fluoro-L-dopa. J. Nucl. Med..

[18] Lemaire C., Libert L., Franci X., Kuci S., Giacomelli F., Genon J.-L., Luxen A. (2015). Automated production at the curie level of no carrier-added 6-[^18^F]fuoro-L-dopa and 2-[^18^F]fuoro-L-tyrosine on a FASTlab synthesizer. J. Label. Comp. Radiopharm..

[19] Krasikova R.N., Zaitsev V.V., Ametamey S.M., Kuznetsova O.F., Fedorova O.S., Mosevich I.K., Belokon Y.N., Vyskočil Š., Shatik S.V., Nader M. (2004). Catalytic enantioselective synthesis of ^18^F-fluorinated α-amino acids under phase-transfer conditions using (S)-NOBIN. Nucl. Med. Biol..

[20] Pretze M., Franck D., Kunkel F., Foßhag E., Wängler C., Wängler B. (2017). Evaluation of two nucleophilic syntheses routes for the automated synthesis of 6-[^18^F]fluoro-l-DOPA. Nucl. Med. Biol..

[21] Wagner F.M., Ermert J., Coenen H.H. (2009). Three-Step, ‘‘One-Pot’’ Radiosynthesis of 6-fluoro-3,4-dihydroxy-L-phenylalanine by isotopic exchange. J. Nucl. Med..

[22] Meleán J.C., Ermert C., Coenen. H.H. (2015). A three-step radiosynthesis of 6-[^18^F]fluoro-L-meta-tyrosine starting with [^18^F]fluoride. J. Label. Comp. Radiopharm..

[23] Preshlock S., Tredwell M., Gouverneur V. (2016). ^18^F-Labeling of arenes and heteroarenes for applications in positron emission tomography. Chem. Rev..

[24] Rotstein B.H., Wang L., Liu R.Y., Patteson J., Kwan E.E., Vasdev N., Liang S.H. (2016). Mechanistic studies and radiofluorination of structurally diverse pharmaceuticals with spirocyclic iodonium(**III**) ylides. Chem. Sci..

[25] Zlatopolskiy B., Zischler J., Urusova E.A., Endepols H., Kordys E., Frauendorf H., Mottaghy F.M., Neumaier B. (2015). A Practical One-Pot Synthesis of Positron Emission Tomography (PET) Tracers via Nickel-Mediated Radiofluorination. Chem. Open.

[26] Tredwell M., Preshlock S.M., Taylor N.J., Gruber S., Huiban M., Passchier J., Mercier J., Genicot C., Gouverneur V. (2014). A general copper-mediated nucleophilic ^18^F-luorination of arenes. Angew. Chem. Int. Ed..

[27] Wright J.S., Kaur T., Preshlock S., Tanzeyl S.S., Winton W.P., Sharninghausen L.S., Wiesner N., Brooks A.F., Sanford M.S., Scott P.J.H. (2020). Copper-mediated late-stage radiofuorination: Five years of impact on preclinical and clinical PET imaging. Clin. Transl. Imaging.

[28] Zlatopolskiy B.D., Zischler J., Krapf P., Zarrad F., Urusova E.A., Kordys E., Endepols H., Neumaier B. (2015). Copper-Mediated Aromatic Radiofluorination Revisited: Efficient Production of PET Tracers on a Preparative Scale. Chem. Eur. J..

[29] Preshlock S., Calderwood S., Verhoog S., Hienzsch A., Cailly T., Schedler M., Mollitor J., Hoepping A., Genicot C., Tredwell M. (2016). Enhanced copper-mediated ^18^F-fluorination of aryl boronic esters provides eight radiotracers for PET applications. Chem. Commun..

[30] Zischler J., Kolks N., Modemann D., Neumaier B., Zlatopolskiy B.D. (2017). Alcohol-Enhanced Cu-Mediated Radiofluorination. Chem. Eur. J..

[31] Antuganov D., Zykov M., Timofeev V., Timofeeva K., Antuganova Y., Fedorova O., Orlovskaya V., Krasikova R. (2019). Copper-mediated radiofluorination of aryl pinacolboronate esters: A straightforward protocol using pyridinium sulfonates. Eur. J. Org. Chem..

[32] Orlovskaya V., Fedorova O., Kuznetsova O., Krasikova R. (2020). Cu-Mediated Radiofluorination of Aryl Pinacolboronate Esters: Alcohols as Solvents with Application to 6-L-[^18^F]FDOPA Synthesis. Eur. J. Org. Chem..

[33] Zlatopolskiy B.D., Zischler J., Schäfer D., Urusova E.A., Guliyev M., Bannykh O., Endepols H., Neumaier B. (2018). Discovery of 7-[^18^F]Fluorotryptophan as a Novel Positron Emission Tomography (PET) Probe for the Visualization of Tryptophan Metabolism in Vivo. Med. Chem..

[34] Craig A., Kolks N., Urusova E.A., Zischler J., Brugger M., Endepols H., Neumaier B., Zlatopolskiy B.D. (2020). Preparation of labeled aromatic amino acids via late-stage ^18^F-fluorination of chiral nickel and copper complexes. Chem. Commun..

[35] ICH Guideline of Elemental Impurities Q3D. https://database.ich.org/sites/default/files/Q3D-R1EWG_Document_Step4_Guideline_2019_0322.pdf.

