# Supplementary material for: Production of 6-l-[18F]Fluoro-m-tyrosine in an Automated Synthesis Module for 11C-Labeling
Source: Molecules. 2021 Sep 13;26(18):5550. doi: 10.3390/molecules26185550 (PMC8468244; doi:10.3390/molecules26185550)
Supplement: Supplementary file 1 [file molecules-26-05550-s001.zip › molecules-1349710-supplementary.pdf]

Supporting information to

Production of 6-L-[^{18}F]fluoro-*m*-tyrosine in an automated synthesis module for ^{11}C-labeling

V. V. Orlovskaya[1*], O. S. Fedorova[1], O. F. Kuznetsova[1], A. S. Craig[2,3*], B. Neumaier[2,3,4#], R. N. Krasikova[1#], B. D. Zlatopolskiy[2,3,4]

[1]*N.P.Bechtereva Institute of the Human Brain, 197376 St.-Petersburg, Russia;*
[2]*Institute of Neuroscience and Medicine, INM-5: Nuclear Chemistry, Forschungszentrum Jülich GmbH, 52425 Jülich, Germany;*
[3]*Institute of Radiochemistry and Experimental Molecular Imaging, University Clinic Cologne, 50937 Cologne, Germany;*
[4]*Max Planck Institute for Metabolism Research, 50931 Cologne, Germany*

Block **I**. Radiofluorination of Bpin-substituted chiral Ni complex **4** and hydrolysis of radiolabeled intermediate [^{18}F]**5:** radio-HPLC traces of the crude reaction mixtures

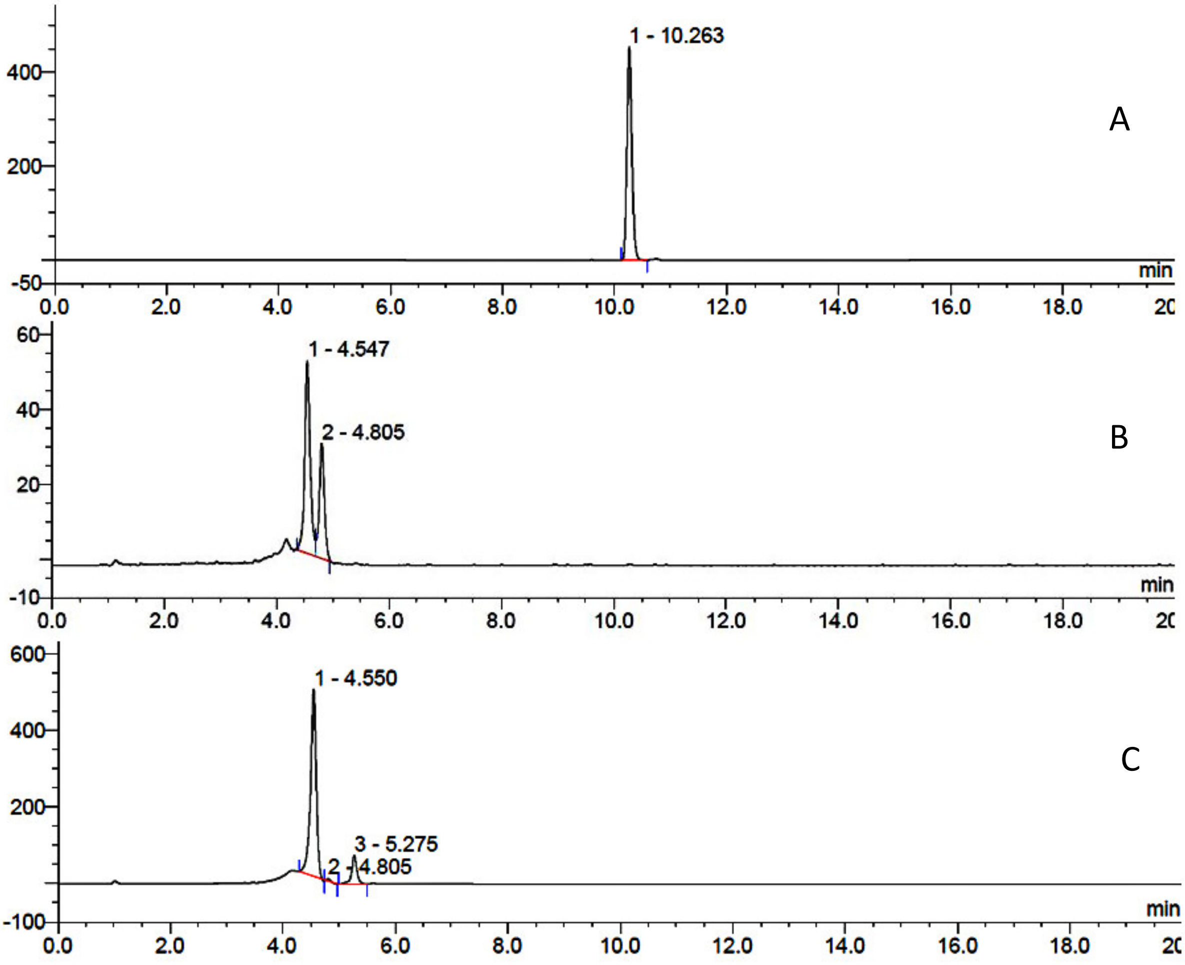

(**A**) radiofluorination of **4** (preparation of [^{18}F]**5**); (**B**) hydrolysis of [^{18}F]**5** with 1.5 N HCl in 50% MeOH; (**C**) hydrolysis of **5** with 0.5 N HCl in 50% MeOH.

HPLC conditions: column: XBridge® C18 5 µm, 150×4.6 mm (Waters, USA); gradient: 0–2 min: 2% MeCN (0.1% TFA), 2–13 min: 2–90% MeCN (0.1% TFA); flow rate: 2.0 mL/min; R_t of [^{18}F]**5**: 10.3 min; R_t of 6-L-[^{18}F]FMT: 4.6 min.

Block **II.** Quality control of 6-L-[^{18}F]FMT (HPLC traces)

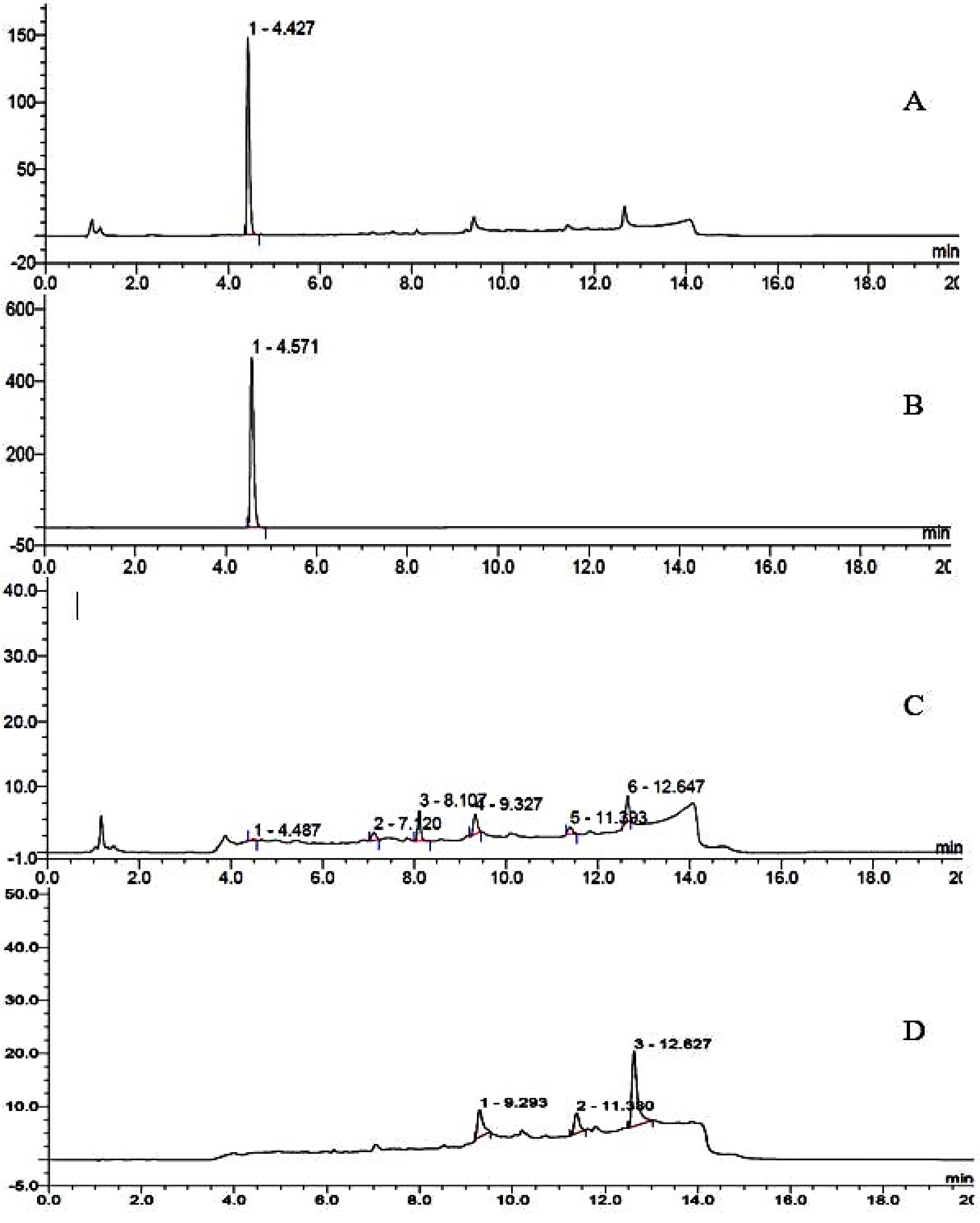

Quality control of 6-L-[^{18}F]FMT: (**A**) 6-DL-FMT, UV 254 nm; (**B**) 6-L-[^{18}F]FMT, radioactivity channel; (C) 6-L-[^{18}F]FMT , UV 254 nm; (**D**) injection of the starting eluent [2% MeCN (0.1%

TFA)], UV 254 nm. The diffuse peak at 3.8–14.0 min and peaks at 3.9; 4.5; 7.2; 8.1; 9.3; 11.4 and 12.6 min (UV 254 nm) are also visible by blank injection.

HPLC conditions: column: XBridge® C18 5 µm, 150×4.6 mm (Waters, USA); gradient: 0–2 min: 2% MeCN (0.1% TFA), 2–13 min: 2–90% MeCN (0.1% TFA); flow rate: 2.0 mL/min; R_t of 6-L-[^{18}F]FMT: 4.6 min.

Block **III**. Control of enantiomeric purity of 6-L-[^{18}F]FMT

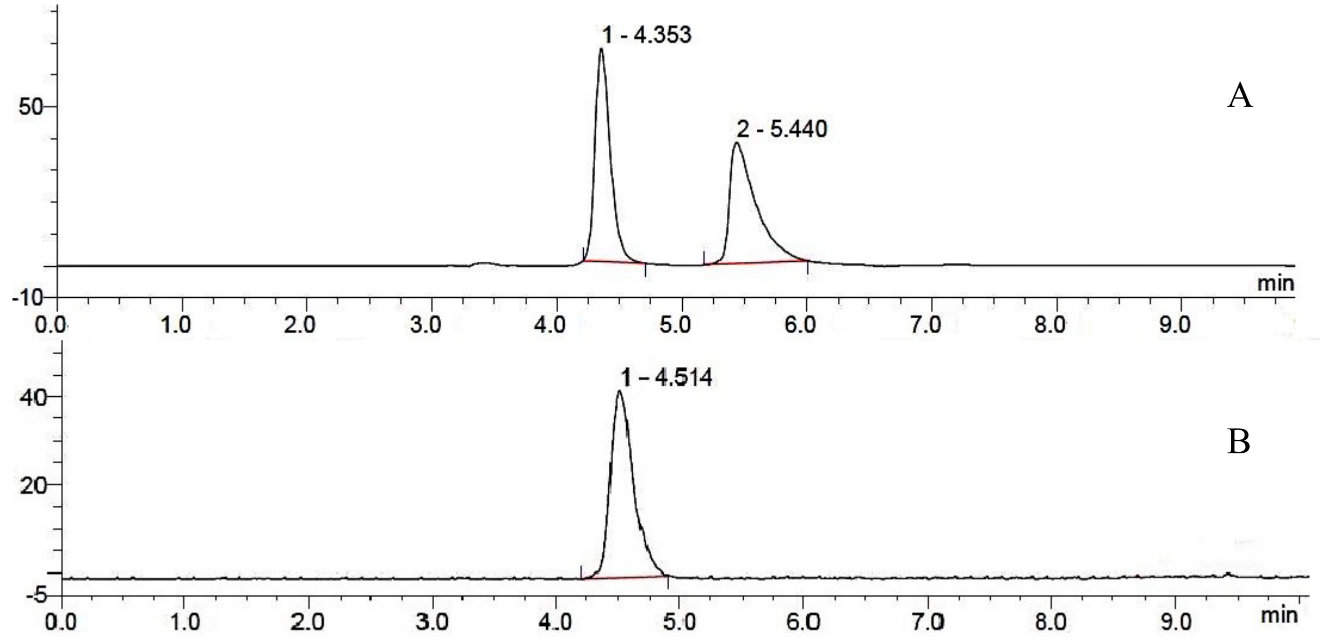

Determination of enantiomeric purity of 6-L-[^{18}F]FMT by chiral HPLC: (**A**) 6-DL-FMT, UV trace (λ=254 nm); peak 1 - 6-L-FMT; peak 2 - 6-D-FMT; (**B**) 6-L-[^{18}F]FMT, radioactivity trace. HPLC conditions: column Chirobiotic T 150×4.6 mm (Astec, USA); eluent: 10% EtOH (0.1% Et_3NOAc); flow rate: 1.0 mL/min.

Block **IV**. **Determination of molar activity (A_m) of 6-L-[^{18}F]FMT**

Determination of molar activity of 6-L-[^{18}F]FMT

The A_m was calculated by dividing the amount of 6-L-[^{18}F]FMT (at the EOS; in GBq) by the amount of 6-L-[^{19}F]FMT (in µmol). The peak areas were determined by HPLC under the conditions described in the caption to Fig. 3. The carrier amount was calculated according to the calibration curve in the concentration range from 0.5 µg/mL to 500 µg/mL.

In the most preparations of 6-L-[^{18}F]FMT, the peak of 6-FMT (carrier) was below the limit of detection. The data from two batches of 6-L-[^{18}F]FMT used to calculate molar activity are presented below.

Batch 1:

Concentration of 6-FMT in the injected sample: 1.2 µg/mL
Batch volume: 4 mL
Mass of 6-FMT in the whole batch: 4.8 µg
Molar mass of 6-FMT = 198
Carrier amount: 0.026 µmol/batch
Batch activity: 3.26 GBq
Molar activity: 125 GBq/µmol

Batch 2:
Concentration of 6-FMT in the injected sample: 0.60 µg/mL
Batch volume: 3.95 mL
Mass of 6-FMT in the whole batch: 2.4 µg
Molar mass 6-FMT = 198
Carrier amount: 0.013 µmol/batch
Batch activity: 3.3 GBq
Molar activity: 253 GBq/µmol
